# Complex Surgical Management of Extensive Chest-Wall Desmoid Fibromatosis

**DOI:** 10.7759/cureus.71670

**Published:** 2024-10-16

**Authors:** Jeffrey Jang, Kathryn Cavallo, Juliet Lee

**Affiliations:** 1 General Surgery, George Washington University, Washington, DC, USA

**Keywords:** chest wall tumour, complex surgical management, desmoid-type fibromatosis, desmoid tumors, reconstructive surgery

## Abstract

Desmoid fibromatosis (DF) is a rare tumor that arises from fibroblasts and myofibroblasts and typically presents on the trunk and limbs. While metastasis of fibromatosis is exceedingly uncommon, DF can spread rapidly to adjacent tissue. Its aggressive nature and frequent recurrence pose challenges for treatment, often requiring a multidisciplinary approach. This case represents the multidisciplinary workup for the identification and management of a rare presentation of desmoid fibromatosis involving the breast. It also discusses the post-surgical follow-up of the patient after a complex resection and reconstruction.

We report a case of a patient treated at George Washington University Hospital, Washington, DC. A 26-year-old woman presented with left chest pain. Physical examination revealed an approximately 12-13 cm, ill-defined, immobile mass arising from the left breast. An MRI demonstrated a 9.5 cm spiculated mass involving the left pectoralis major muscle and underlying costal cartilage junctions. The tumor extended deep to the rib margin and along the anterior margin of the pleura. The mass was resected en bloc in a joint case with thoracic and general surgery and reconstructed with a bilateral bi-pedicled, muscle-sparing, transverse rectus abdominis myocutaneous free flap with plastic surgery. Pathology demonstrated desmoid fibromatosis.

In conclusion, desmoid tumors of the breast represent a very rare breast neoplasm that can imitate other breast masses, both benign and malignant. Surgery remains an important treatment option and may require coordination and complex surgical planning between various surgical subspecialties.

## Introduction

Desmoid tumors are known to be difficult to manage, with high rates of recurrence. Desmoid fibromatosis (DF) can be classified as intra-abdominal and extra-abdominal. They most commonly affect younger patients, particularly females, and often present on the trunk or extremities. Desmoid tumors of the breast are rare [1-3]. While general management has shifted more toward active surveillance and medical treatment, surgical resection may be required for large or invasive tumors. Breast DF poses a unique surgical challenge, as it requires balancing adequate tumor resection, which may be in close proximity to critical thoracic structures, with acceptable cosmetic and functional outcomes.

## Case presentation

A 26-year-old woman with a history of a resected breast mass presented with left chest pain. She was gravida 2, para 2, with recent lactation. The patient complained of persistent, severe pain in the left chest, worsened with movement, that had been present for several months. She reported symptoms exclusively in the left chest with no radiation. The patient was an immigrant with a history of breast mass resection abroad three years prior but was unable to provide details on the extent of the surgery or characteristics of the resected mass.

Physical examination revealed an approximately 12-13 cm, ill-defined, immobile mass arising from the left breast that was moderately tender to palpation. It was associated with limited abduction of the left upper extremity due to pain. There were no overlying skin changes. There was milky nipple discharge consistent with the patient’s lactation from her recent pregnancy.

The patient was evaluated by general, cardiothoracic, and plastic surgery teams at an urban academic hospital. Initial physical exam findings of the left breast mass suggested soft tissue sarcoma rather than a breast tissue origin.

**Figure 1:**
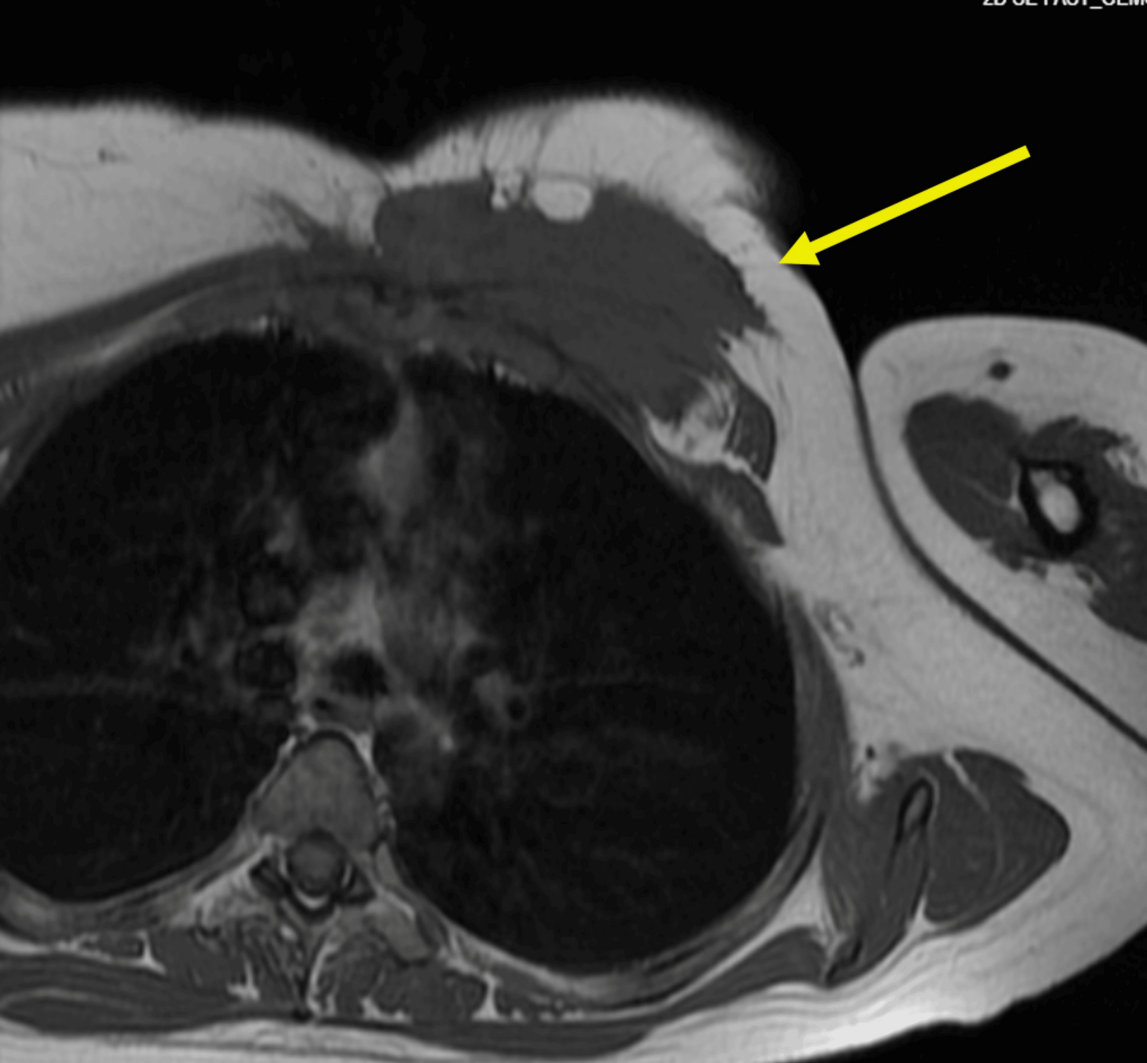
MRI of left chest wall mass involving the left pectoralis major muscle, underlying costal cartilage junctions, and abuts the anterior pleura.

**Figure 2:**
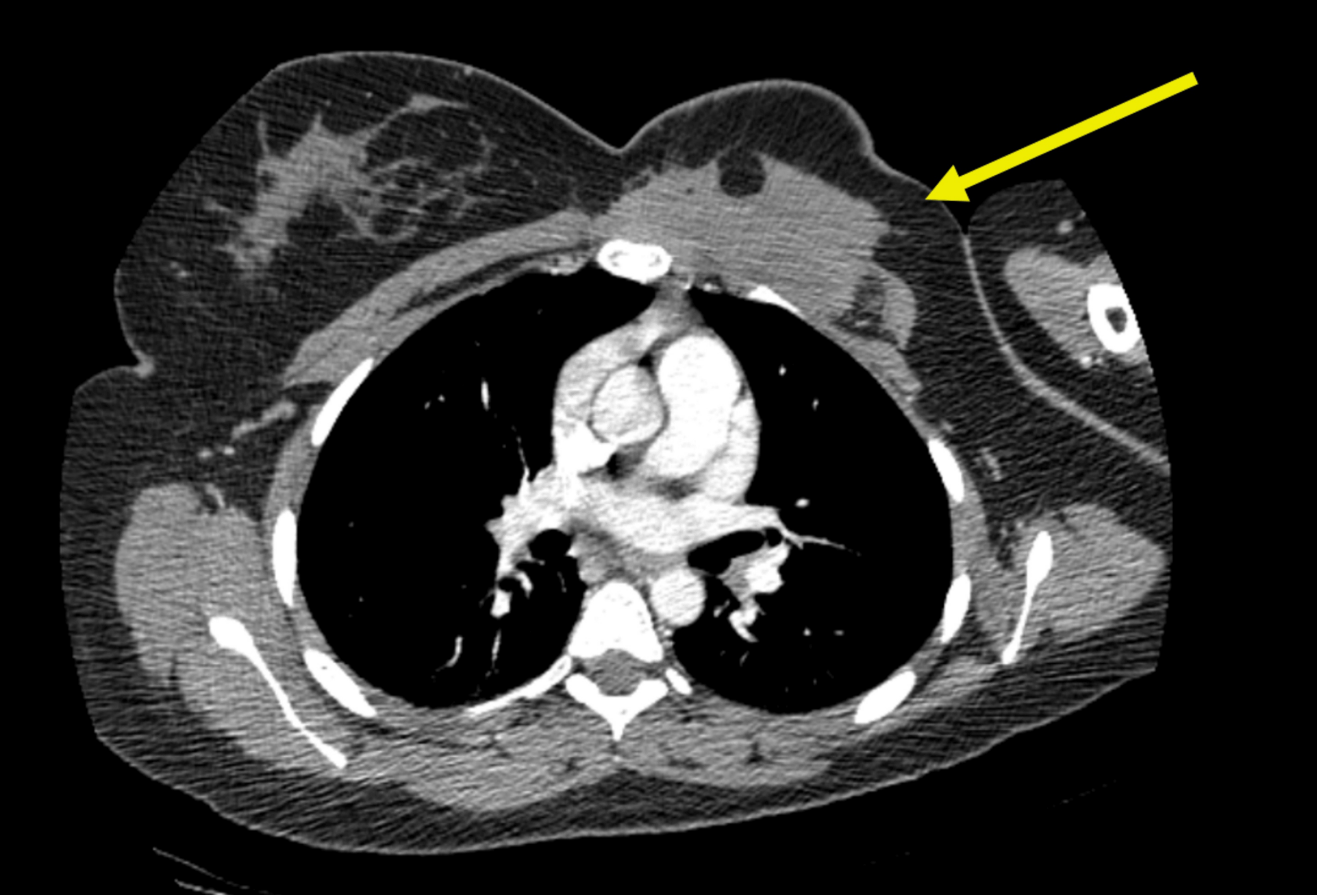
CT Thorax with contrast in the transverse plane which demonstrates a partially enhancing spiculated mass in the left anterior chest wall with associated skin thickening and irregularity.

**Figure 3:**
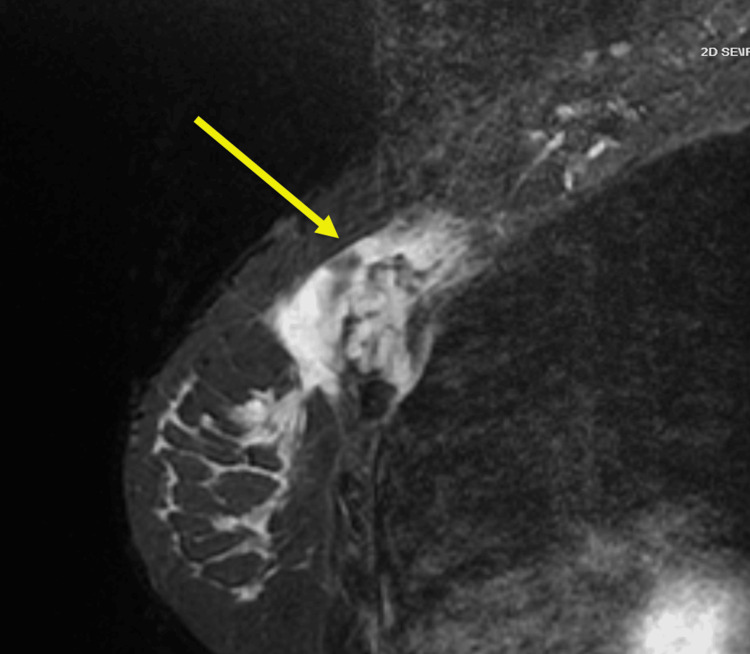
CT Thorax with contrast in the sagittal plane which demonstrates a partially enhancing spiculated mass in the left anterior chest wall with associated skin thickening and irregularity.

The patient underwent MRI, which demonstrated a 9.5 cm spiculated mass involving the left pectoralis major muscle and underlying costal cartilage junctions (Figure 1). The tumor extended deep to the rib margin and along the anterior margin of the pleura. The mass also abutted the breast parenchyma. A subsequent computed tomography (CT) scan with contrast showed similar findings, with the mass abutting the sternomanubrial joint and invading the chest wall at the left second intercostal space (Figures 2, 3).

An ultrasound was performed for a guided core-needle biopsy, which revealed a large hypoechoic mass with septations and debris. The core-needle biopsy indicated fibrous tissue with focal acute and chronic inflammation.

The mass was resected en bloc in a joint procedure with cardiothoracic and general surgery. The procedure included a left total mastectomy with resection of ribs 2-3, sternum, and parietal pleura. The soft tissue deficit was covered with Gore-Tex mesh. The internal mammary arteries were identified bilaterally and clipped for use by plastics in flap anastomosis. A chest tube was placed due to pleural resection. Plastic surgery then completed a bilateral bi-pedicled, muscle-sparing, transverse rectus abdominis myocutaneous (MS-TRAM) free flap. SPY-indocyanine green (ICG) angiography was used intraoperatively and demonstrated excellent perfusion. OviTex mesh was used to reinforce the abdominal wall donor site in an underlay fashion.

**Figure 4:**
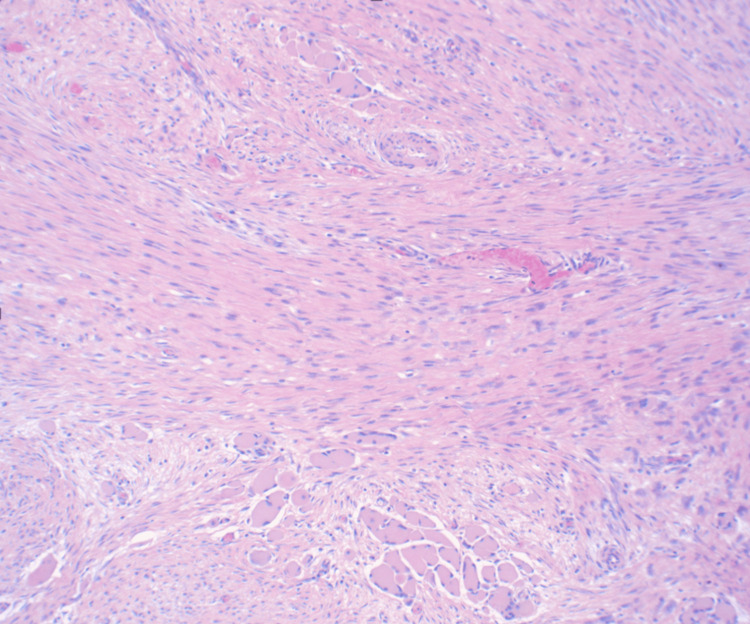
characteristic hematoxylin and eosin (H&E) slide demonstrating desmoid fibromatosis with characteristic spindle cell proliferation with collagenous stroma background.

**Figure 5:**
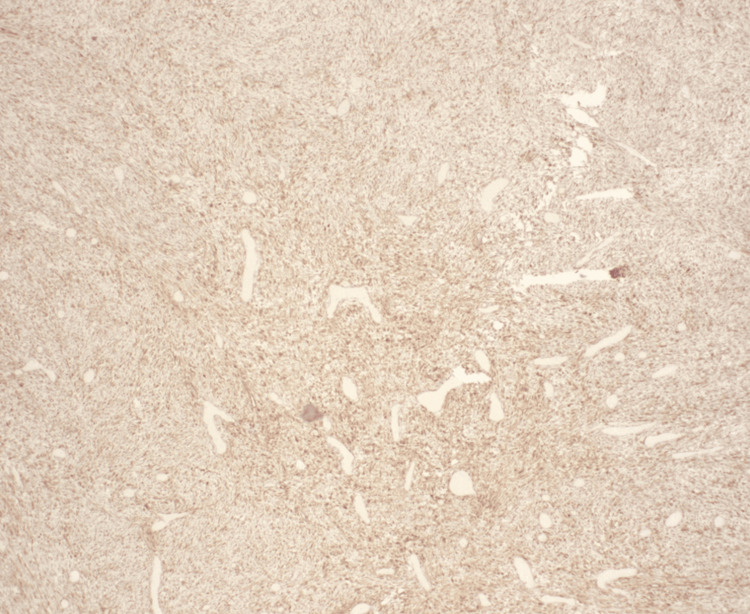
Immunohistochemical stain diffusely positive for beta catenin, a characteristic tumor marker of desmoid neoplasm.

Pathology reports of the surgical specimens indicated a desmoid fibromatosis tumor invading the muscle and sternal cortical bone (Figure 4). The tissue sample stained positive for beta-catenin (Figure 5). Superior and medial soft tissue margins were focally involved by the tumor, including the involvement of the dermis. The left breast skin and nipple were evaluated with benign findings, negative for fibromatosis.

The patient tolerated the procedure well and remained intubated in the ICU. She was extubated on postoperative day (POD) 1. The chest tube was removed on POD 5. She was discharged to home on POD 6. The patient presented to the plastic surgery clinic several weeks later with breakdown of the abdominal incision and was readmitted for a surgical site infection, requiring intravenous antibiotics, operative debridement, and wound vac placement. She received chest wall radiation to a dose of 5400 cGy in 30 fractions starting six weeks postoperatively. Approximately nine months postoperatively, she experienced soft tissue radionecrosis of the left breast, complicated by chest wound infection. This required surgical debridement with partial mesh infection, wound vac placement, and oral antibiotics.

## Discussion

Demographic details

DF tends to affect younger patients, with a typical age range from 22 to 76 years. It more commonly occurs in women, but men can also be affected [4].

Medical history

While it is unclear which risk factors play a role in the development of DF of the breast, recurring backgrounds have been identified. Trauma to the breast, whether due to disease or surgical intervention, may be connected to DF. One study of 32 patients showed that 25% of DF patients had a previous diagnosis of breast cancer, and 44% had previous breast surgery. Numerous case reports have outlined DF in the context of a history of silicone breast implants, although no formal association has been described [5, 6].

Breast DF more commonly affects women, and there may be an association with pregnancy and hormonal influences in the initial development and growth of tumors. However, this association has yet to be formalized [4]. There does not appear to be much evidence or case reports linking desmoid fibromatosis of the breast with other familial conditions predisposing to desmoid tumors, such as Gardner syndrome or familial adenomatous polyposis (FAP).

Symptoms

Generally, DF of the breast initially presents as a painless palpable mass. Skin findings, such as dimpling and color changes, are variable. The initial presentation of DF can overlap with more common breast masses, such as carcinomas and fibroadenomas. The similarity in patient symptoms and exam findings can pose a challenge in the identification of DF.

Evaluation and diagnosis

Evaluation of DF tumors is based on imaging and core-needle biopsy. Imaging is often non-specific for DF and can imitate other malignant and benign breast masses. While some cases describe MRI as the gold standard for diagnosis, other cases have incorporated mammography, ultrasonography, and computerized tomography. On mammography, DF appears as an irregular, star-shaped spiculated mass without calcifications [3]. Initial evaluation with mammography can vary, but one study showed that 92% of DF patients who underwent mammography had a Breast Imaging-Reporting Data Systems (BI-RADS) score of 4 [7]. Given that DF can affect patients younger than 45, the limitation of mammography in evaluating dense breast tissue should be taken into consideration [8]. On ultrasonography, DF exhibits a hypoechoic pattern with irregular margins. Soft-tissue and chest wall involvement is best visualized with MRI and CT. MRI is the preferred method of imaging in the preoperative setting to define the extent of invasion of breast DF [1,7].

Fine-needle aspiration is generally not useful in the workup of fibromatosis [9]. Core-needle biopsy can be performed prior to surgery to confirm DF, but there are limitations given the small biopsy volume, which can mimic low-grade sarcomas [8,9]. Diagnosis is confirmed with histological evaluation of surgical specimens. A hallmark finding of DF is low to moderate cellularity with no increase in mitotic activity. Classically, DF consists of non-encapsulated, bland-looking spindle cells that are often positive for beta-catenin [3,9].

Breast DF does not have an accepted staging system, and metastasis is not expected. Prognosis is based on individual clinical factors [9,10].

Treatment/intervention and outcomes

Treatment options for DF of the breast have historically been challenging, with high rates of recurrence. The available options include active surveillance (AS), medical management, surgical management, and radiation therapy. Generally, the optimal treatment plan is individualized for each patient and should involve discussion with a multidisciplinary team [11].

The Desmoid Tumor Working Group classifies DF of the breast as a favorable location compared to other sites of desmoid tumors. For patients without symptoms, AS is recommended if DF does not progress. AS consists of an initial MRI or CT with repeated imaging every three to six months. AS has shown a 63% two-year event-free survival (EFS) in DF at favorable locations, which is similar to surgical management. Active treatment is not recommended unless disease progression or burden is demonstrated on two assessments. If DF progresses, medical and surgical management options are recommended [11].

In terms of medical management, it is important to note that most literature evaluates pharmacological efficacy against all desmoid tumor locations, not specifically breast DF. Many conclusions include patients with more aggressive forms of desmoid tumors, as well as patients with FAP. Medical treatments for desmoid tumors include nonsteroidal anti-inflammatory drugs (NSAIDs), hormone therapy, immunotherapy, and cytotoxic chemotherapy.

Hormone therapy for the treatment of desmoid tumors has been studied to varying degrees and is described in individual case reports. Tamoxifen and toremifene are the most common hormone therapies used to treat DF. The response to antiestrogen therapy is mixed, with successes more often associated with individual cases rather than larger studies. In one larger study involving 25 patients, tamoxifen and NSAIDs showed higher rates of stable disease or partial regression compared to surgery. However, none of the patients in the study had DF of the breast [12,13]. There is a need for more studies evaluating the response of breast DF to antiestrogen medical therapy. DF of the breast tends to be less aggressive than other desmoid tumors, including those associated with FAP. It is difficult to extrapolate the efficacy of antiestrogen therapies across different anatomical sites.

Tyrosine-kinase inhibitors (TKIs) are also used to treat desmoid tumors, including breast DF. Sorafenib, pazopanib, and imatinib are the most commonly used TKIs, though recent trends and guidelines have shifted away from imatinib [11]. Sorafenib has recently shown efficacy in recurrent and progressive tumors compared to placebo. It has been used to treat breast DF with a successful reduction in tumor volume [5,14,15].

Cytotoxic chemotherapy may be appropriate depending on the characteristics of the DF. Generally, it is not a first-line medical therapy due to associated toxicities, but it does have known activity against desmoid tumors, with different strategies for its use. Low-dose methotrexate combined with vinblastine has been studied to reduce toxicity. Responses have shown favorable progression-free survival [16]. Conventional-dose chemotherapy, with agents such as anthracyclines, has also been used, potentially offering a faster response rate and shorter treatment times. Cytotoxic chemotherapy has a role in the medical management of DF of the breast, particularly for patients with rapidly growing DF or those unresponsive to other medical therapies [9,10].

Surgery remains an important treatment option, especially in symptomatic patients with large areas of invasion. It is also indicated for patients who have undergone AS and medical management with continued disease progression or for those who have had prior surgery and experienced disease recurrence. When surgery is indicated, the conventional goal is to achieve negative margins to reduce the risk of recurrence. In a larger study of chest wall DF, recurrence was evaluated over 21 years. Of the patients with positive margins, 89% experienced recurrence, while 18% of patients with negative margins had recurrence [17]. Surgery with negative margins for breast DF is also associated with fewer recurrences; however, even in this group, recurrence is common. Other factors, beyond surgical margins, likely affect recurrence rates, calling into question whether negative margin resection should remain the definitive surgical goal. For example, younger age and larger tumor size at the time of surgery may be associated with a higher rate of recurrence [4,18].

Surgical options for breast DF should offer an individualized approach, involving the appropriate teams to achieve negative resection margins and prevent recurrence, while also ensuring adequate tissue coverage when necessary. Surgery can significantly impact the patient both physically and psychologically, so balancing the patient's goals regarding acceptable risks of recurrence with quality of life after surgery is essential.

Radiotherapy can be used following surgery. Some studies suggest that radiation provides a modest reduction in absolute risk of recurrence after surgery. However, there is limited evidence suggesting clear benefits from radiation. Guidelines on desmoid tumor treatment, though not specific to breast DF, indicate that adjuvant radiation may be beneficial for recurrent disease [11].

## Conclusions

DF of the breast presents a challenge to manage. It is a rare disease that can mimic other, more common breast malignancies. Its treatment is unique in that the first-line management is active surveillance. When surgery is indicated, the goal is to achieve negative margins. This poses a surgical challenge for breast DF, particularly when considering the tumor’s proximity to critical thoracic structures and the need for reconstruction strategies. It is therefore essential to consider individual patient characteristics and preferences regarding both surgical intervention and ongoing surveillance. Surgical management of DF of the breast has been described in individual case reports, using many of the techniques outlined by the Desmoid Tumor Working Group. However, additional research is needed to study breast-specific desmoid tumors and to better characterize novel surgical approaches that maintain acceptable cosmetic and functional outcomes for patients.
